# An Unusual Presentation of Candidal Onychomycosis: A Case Report

**DOI:** 10.7759/cureus.43222

**Published:** 2023-08-09

**Authors:** Fawaz H Aljehani, Razan Alluhaibi, Omar S Alhothali, Sarah M Fageeh, Ghaida A Al Ahmadi, Rana Z Malyani

**Affiliations:** 1 Dermatology, King Abdulaziz Hospital, Makkah, SAU; 2 Faculty of Medicine, Umm Al-Qura University, Makkah, SAU

**Keywords:** type 2 diabetes, fungal infection, nails, hyperkeratosis, onychomycosis

## Abstract

Onychomycosis can present with various manifestations such as subungual hyperkeratosis, onycholysis, and nail plate destruction. Here we present a case of a 61-year-old African male with a known case of type 2 diabetes mellitus on insulin. He worked as a mechanic and presented with nail changes that started four months prior to presentation and worsened over time, mainly affecting the fingernails of bilateral hands. On examination, there was yellowish to greenish discoloration with very extensive hyperkeratosis of skin around the fingers and nails that caused avulsion of nails. Swab and culture showed Candida albicans +3. Nail and skin biopsy showed bacterial colony with fungal hypha. The patient showed marked improvement after receiving oral fluconazole 300 mg weekly for three months.

## Introduction

Onychomycosis, a fungal nail infection, is a common medical condition in adults. It has recently been discovered that onychomycosis affects approximately 20% of those aged 40 to 60 years [1]. Other risk factors for onychomycosis include frequent fungal exposure, empirical antibiotic medication, HIV infection, and immunosuppressive drug therapy [2]. Other contributory factors comprise diabetes mellitus, peripheral vascular disease, chronic smoking, prolonged or repetitive water immersion, and trauma to aged nails [2].

The most common anthropophilic dermatophytes that cause onychomycosis are *Trichophyton rubrum* and *Trichophyton mentagrophytes* var. interdigitale. Molds that are not dermatophytes, such as* Scopulariopsis brevicaulis* and *Aspergillus* spp., can cause onychomycosis as primary pathogens or as contaminant agents and secondary pathogens [3]. The estimated worldwide prevalence of non-dermatophyte mold (NDM) onychomycosis is 10%-15% [4]. The third source of nail fungal infection is yeasts, such as *Candida albicans* and *Candida parapsilosis*, which only manifest when predisposing conditions, including immunosuppression and diabetes, are present [1].

The most prevalent presentation of dermatophyte nail infection is distolateral subungual onychomycosis. Toenails are more commonly affected than fingernails. The fungus invades the nail and nail bed by penetrating the distal or lateral margins. The affected nail thickens and discolors, with different degrees of onycholysis (separation of the nail plate from the nail bed). Invasion through the proximal margin, which is embedded within the proximal nail fold, is more prevalent in those with immunodeficiency (proximal subungual onychomycosis) [5]. Progression of the disease can lead to variants and overlap of these presentations.

Candidal onychomycosis affects fingernails more frequently than toenails, with *Candida* species accounting for approximately half of all fingernail-related onychomycoses [1]. We report a rare case of onychomycosis presented as a very extensive hyperkeratosis of skin around the fingernails in a 61-year-old male patient caused by *Candida albicans*.

## Case presentation

**Figure 1:**
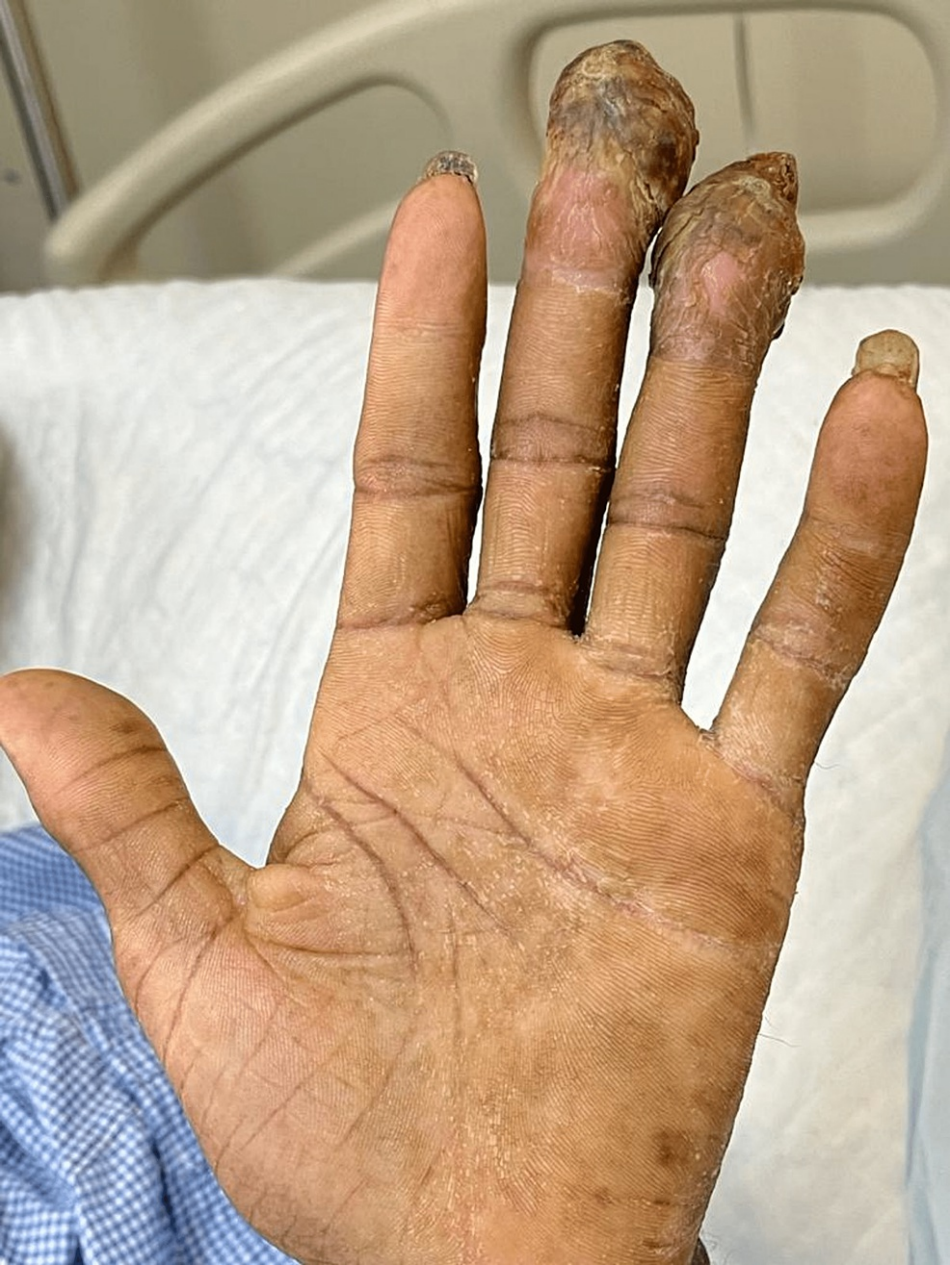
Extensive debris and pus under the nails, with yellowish to greenish discoloration of the nails.

**Figure 2:**
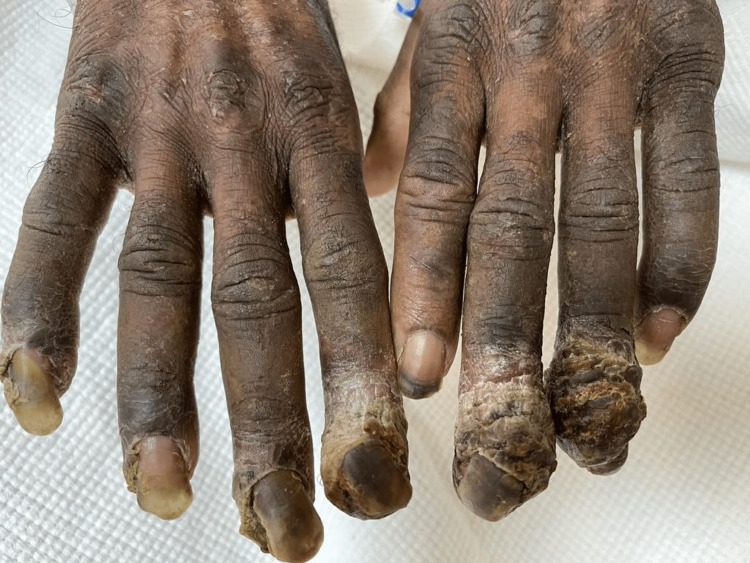
Nails curved and avulsed from the nail bed.

**Figure 3:**
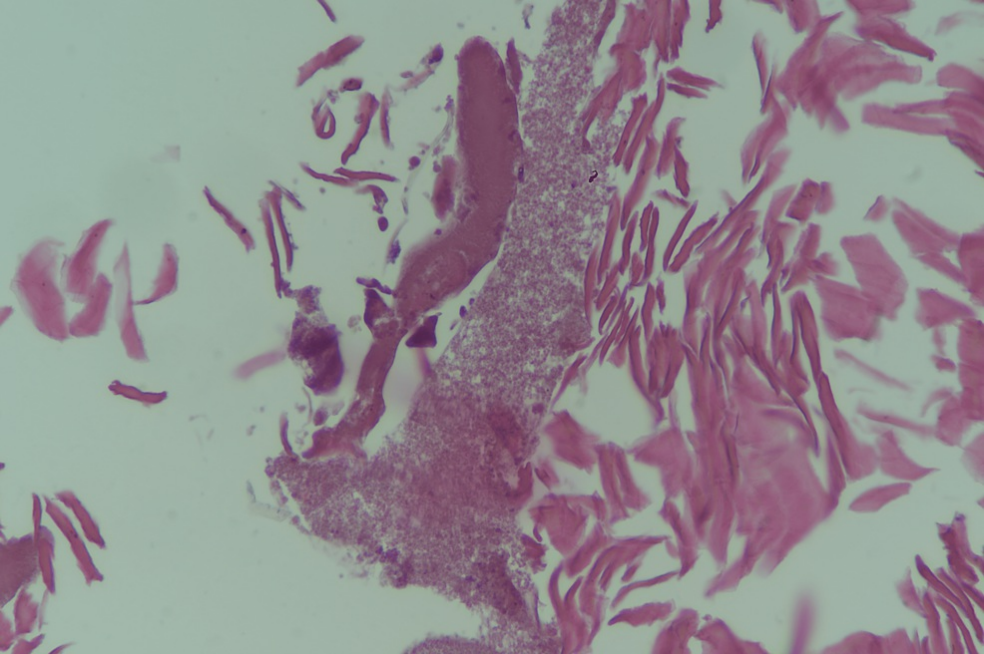
A right middle finger nail bed biopsy showed necrotic tissue with keratin flakes and bacterial colony with fungal hypha.

The patient is a 61-year-old African male with a known case of type 2 diabetes mellitus on insulin. He worked as a mechanic and had a history of necrotizing fasciitis of the left lower leg, for which debridement and grafting had been performed 10 months ago; he now presented with leg cellulitis and nail changes that started four months ago, increasing and worsened over time, mainly of the finger's nails of both hands more than toes nails, associated with pain. The patient denied any trauma, exposure to moisture, smoking, or drug abuse. On examination, there was very extensive hyperkeratosis (oyster shell-like) of skin around the fingernails (thumb, index, and middle fingers of the right hand, and middle and ring fingers of the left hand); the hyperkeratosis was causing avulsion of the nails from the place. The nails curved and detached from the nail bed, with extensive debris and pus under the nails and yellowish-to-greenish discoloration of nails (Figures 1, 2). The toenails were more mildly affected than the fingernails. The soles and palms show a picture of retention hyperkeratosis, with no mucus membrane involvement. Labs were negative for HIV serology, with normal thyroid function test and normal X-ray of the bilateral hands. A swab for bacterial culture from the pus, fungal culture for the nail, and debris showed Candida albicans +3. A right middle finger nail bed biopsy showed necrotic tissue with keratin flakes and bacterial colony with fungal hypha (Figure 3).

**Figure 4:**
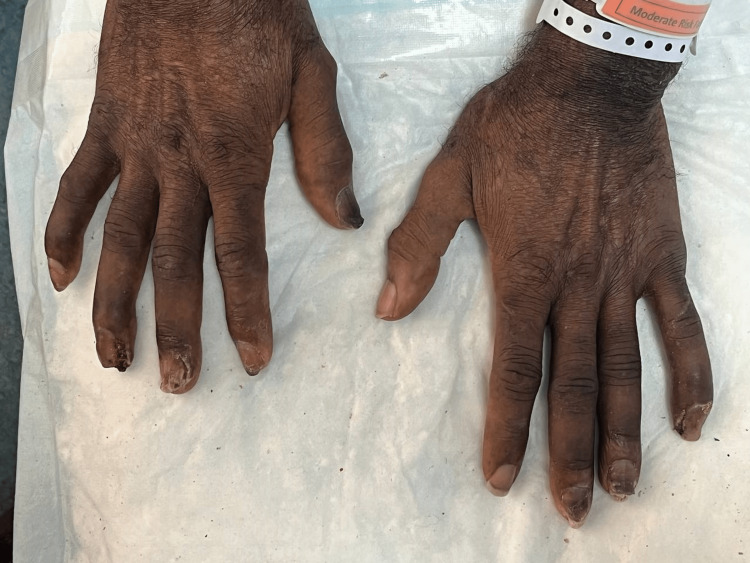
Marked improvement of the nail changes after receiving oral fluconazole 300 mg

The patient received potassium permanganate solution 1/10,000 IU twice a day and salicylic acid ointment twice a day for the palms and soles. No debridement was performed for the infected fingertips. All debris and infected material of the fingertips fall by themselves with topical antiseptic solution and systemic anti-fungal. Follow-up after receiving oral fluconazole 300 mg weekly for three months showed marked improvement in nail changes (Figure 4).

## Discussion

Onychomycosis is a fungal nail infection of the fingernails or toenails that may infect any part of the nail units, and is usually presented with thickening, discolorations, separation from the nail bed, or subungual hyperkeratosis [6]. Diagnosing onychomycosis based on clinical examination alone may be inaccurate. Thus, microscopic examination and culture are essential to confirm the diagnosis and exclude other differential diagnoses [6].

The most common pathogen cause onychomycosis is dermatophytes, with non-dermatophytes representing less commonly [7]. Recently, studies performed in Iran, Colombia, and Italy revealed an increase in NDM cases, while dermatophytes decreased significantly [8-10]. Another study in China reported that the incidence of yeast increased recently, especially *Candida *[11].

In the literature, there are many cases of onychomycosis infected by non-dermatophytes. A 42-year-old male immunocompetent with no co-morbidities had fingernails infection due to non-dermatophytes fungus and presented with discoloration of all fingernails, dystrophic changes, and onycholysis [12]. Another 50-year-old medically free farmer presented with yellowish fingernail discoloration and thickening without associated symptoms. Examination showed distal and lateral subungual onychomycosis with yellowish discoloration and thickened nails. The culture showed yeast-like colonies as features of *Candida* species [11]. But none of these cases presented as our case with extensive hyperkeratosis caused by non-dermatophytes.

Yeast has got more attention recently due to its rising morbidity, particularly the *Candida* species [13]. The previous study reported that the most prevalent species was *Candida albicans*, representing 34.9%, followed by Trichophyton interdigitale [11]. Subungual hyperkeratosis is considered one of the poor prognostic factors in the onychomycosis severity index, which decrease the penetration of antifungal therapy if more than 2 mm in thickness [14].

We reported a rare presentation of candidal onychomycosis with extensive hyperkeratosis, as dermatologists should be aware of its unusual presentation. We want to increase awareness by developing tools to assess onychomycosis severity to guide proper patient treatment and prevent further complications affecting the quality of life.

## Conclusions

Onychomycosis may manifest with subungual hyperkeratosis or discoloration. Many conditions can mimic onychomycosis, especially the candida type, which affects fingernails more than toenails. Herein, we present a rare presentation caused by candidal onychomycosis. Thus, proper management is important to improve the psychosocial and physical aspects of the patients.
